# Antibiotic Degradation via Fenton Process Assisted by a 3-Electron Oxygen Reduction Reaction Pathway Catalyzed by Bio-Carbon–Manganese Composites

**DOI:** 10.3390/nano14131112

**Published:** 2024-06-28

**Authors:** Edgar Fajardo-Puerto, Abdelhakim Elmouwahidi, Esther Bailón-García, María Pérez-Cadenas, Agustín F. Pérez-Cadenas, Francisco Carrasco-Marín

**Affiliations:** 1 UGR-Carbon, Materiales Polifuncionales Basados en Carbono, Dpto. de Química Inorgánica, Unidad de Excelencia de Química Aplicada a Biomedicina y Medioambiente, Universidad de Granada (UEQ-UGR), 18071 Granada, Spain; edgarf1994@correo.ugr.es (E.F.-P.); estherbg@ugr.es (E.B.-G.); mariaperez@ccia.uned.es (M.P.-C.); afperez@ugr.es (A.F.P.-C.); fmarin@ugr.es (F.C.-M.); 2 Dpto. Química Inorgánica y Química Técnica, Facultad de Ciencias, Universidad Nacional de Educación a Distancia (UNED), Av. de Esparta s/n, Las Rozas de Madrid, 28232 Madrid, Spain

**Keywords:** olive wastewater, bio-carbon–manganese composite, ORR, electro Fenton

## Abstract

Bio-carbon–manganese composites obtained from olive mill wastewater were successfully prepared using manganese acetate as the manganese source and olive wastewater as the carbon precursor. The samples were characterized chemically and texturally by N_2_ and CO_2_ adsorption at 77 K and 273 K, respectively, by X-ray photoelectron spectroscopy (XPS) and X-ray diffraction. Electrochemical characterization was carried out by cyclic voltammetry (CV) and linear sweep voltammetry (LSV). The samples were evaluated in the electro-Fenton degradation of tetracycline in a typical three-electrode system under natural conditions of pH and temperature (6.5 and 25 °C). The results show that the catalysts have a high catalytic power capable of degrading tetracycline (about 70%) by a three-electron oxygen reduction pathway in which hydroxyl radicals are generated in situ, thus eliminating the need for two catalysts (ORR and Fenton).

## 1. Introduction

The contamination of water with antibiotics has become a global concern due to the associated risks to both the environment and public health. This includes the emergence of super-resistant bacteria that can cause new diseases and are able to tolerate treatments that were previously effective [1,2]. In general, the most widely used antibiotics worldwide are tetracycline (TC), quinolone, aminoglycoside, macrolide, and sulfonamide [3] TC, due to its low cost and broad antimicrobial spectrum, is used in both humans and animals; however, due to its low metabolization, it has even been found in drinking water [4]. Conventional methods for degrading this type of contaminant, such as biological processes, filtration, coagulation, flocculation, sedimentation, adsorption, and membrane processes, have proven to be inadequate or inefficient. Therefore, it is necessary to explore new processes capable of partially or completely mineralizing these molecules [5,6,7,8,9]

Advanced oxidation processes (AOPs) are a highly regarded option for reducing various persistent pollutants in water due to the generation of hydroxyl radicals (^·^OH) that have a high oxidative capacity (2.8 V vs. RH) capable of partially or completely mineralizing a broad range of pollutants. These radicals are produced on-site when using AOPs, which provides a powerful, alternative method for eliminating persistent pollutants in water [10,11,12]. The electro-Fenton (EF) process is a notable AOP due to its high rate of electro-generated H_2_O_2_, low production of iron sludge, and environmentally friendly nature [13]. In general, the EF process is based on the in situ production of H_2_O_2_ from the oxygen reduction reaction via two electrons (ORR 2e^-^) (Equation (1)) at the cathode, generation of ^·^OH by reacting the Fenton-like catalyst (usually Fe^2+^) with H_2_O_2_ (Equation (2)), and, finally, regeneration of Fe^2+^ in the cathode (Equation (3)) and by interaction with H_2_O_2_ (Equation (4)) [14,15,16,17].

(1) $$O_{2}+2H^{+}+2e^{-}\to H_{2}O_{2}$$

(2) $$H_{2}O_{2}+{Fe}^{2+}\to {Fe}^{3+}+{HO}^{-}+{\;}^{\cdot }OH$$

(3) $${Fe}^{3+}+e^{-}\to {Fe}^{2+}$$

(4) $$H_{2}O_{2}+{Fe}^{3+}\to {Fe}^{2+}+{\;}^{\cdot }OH_{2}+H_{2}O$$

As can be seen, the cathode material is a determining factor in the efficiency of the EF process [18]. So, recently, different types of materials have been used among which noble metals [19,20], metal oxides [21,22], and carbonaceous materials stand out [23,24,25]. However, carbonaceous materials stand out mainly for their low cost, good stability, and abundance [26,27]. Various carbonaceous materials, in order to reduce costs, have been made from different biomasses such as bamboo [28], black soybean [29], the inner layer of torreya grandis [30], waste wood (balsa wood [31], water hyacinth [32], cellulose [33], and, recently, in relation to the circular economy, sewage sludge has been used as a source of biocarbon [34]. However, the main drawback with this type of carbon material is the low activity and slow kinetics [35,36,37], so it is necessary to find a strategy to overcome these limitations and ameliorate the catalytic performances of carbon materials. To this end, the formation of a composite that exploits the advantages of carbon materials will be advantageous for use in electrocatalysis due to the synergistic effect between the metal catalyst and the catalytic support. In addition, the structure, composition, and concentration of the catalyst and support can be precisely modulated by controlling the stoichiometry of the precursors [38,39,40].

In general, doping with transition metals has resulted in a strategy to improve the ORR activity of carbonaceous materials, since, in addition to this, transition metal ions can serve as electron donors to generate ^·^OH from H_2_O_2_ [41]_._ Among the transition metals, MnOx oxides stand out, due to their different applications such as catalysis, energy storage, and, specifically, in electrochemical advanced oxidation processes (EAOP) [42]. MnO_2_ has been shown to have the ability to dissociate H_2_O_2_ to ^·^OH at a faster reaction rate [43]. However, the application of carbonaceous materials doped with Mn as possible bifunctional catalysts for the generation direct of ^·^OH by a three-electron pathway has not yet been studied.

In the present work, a biocarbon obtained from “alpechín” olive mill wastewater generated from the production of olive oil [44] was synthesized through a chemical activation process and treated with Manganese with three different methods and loadings. The synthesized composite materials were evaluated in an environmental remediation process as tetracycline degradation by the electro-Fenton process.

## 2. Experimental

### 2.1. Bio-Carbon–Manganese Composites Preparation

A bio-carbon-denominated CK2 was the base for the development of the composite series. The CK2 was prepared from olive mill wastewater by chemical activation with potassium hydroxide (KOH), the preparation details and textural characterization are reported in previous works [45]. Manganese acetate ((CH_3_COO)_2_Mn·4H_2_O, Sigma Aldrich, St. Louis, MI, USA) was used as a manganese precursor. Different amounts of the manganese acetate were dissolved in water and were added dropwise onto the corresponding amount of bio-carbon.

After impregnation, the prepared mixture was treated by three different methods. The composites were noted as CK2-Mn-X-Y; X corresponds to the method used for the preparation and Y traduces the theoretical manganese percentage.

The first sample was calcined (after impregnation with 10% of manganese) at 330 °C for 1 h under nitrogen gas (300 mL min^−1^) and 1 h under CO_2_ flow. The corresponding sample was noted as CK2-Mn-1-10. The second sample was prepared by adding H_2_O_2_ (25 mL g^−1^ carbon) to the impregnated samples and, after that, the samples were calcined at 330 °C for 2 h under 300 mL min^−1^ of nitrogen. The corresponding composites were noted as CK2-Mn-2-10, CK2-Mn-2-25, and CK2-Mn-2-60. The last sample was prepared by a few modifications of the second method, the impregnated sample was calcined at 330 °C for 2 h under 300 mL min^−1^ of nitrogen before being treated with H_2_O_2_ (25 mL g^−1^ carbon), CK2-Mn-3-10. 

### 2.2. Textural and Chemical Characterization 

The textural characterization was obtained by using Autosorb equipment from Quantachrome system (Boynton Beach, FL, USA). N_2_ adsorption isotherm at −196 °C and CO_2_ at 0 °C were obtained. The B.E.T. and Dubinin–Radushkevich (DR) methods were applied to the nitrogen and carbon dioxide isotherms, respectively, to obtain the apparent surface area, total micropore volume (W_0_ with N_2_), narrow micropore volume (W_0_ with CO_2_), and mean micropore width (L_0_) [46,47,48]. The pore-size distribution (PSD) was determined by applying Quenched Solid Density Functional Theory (QSDFT) to the N_2_ adsorption isotherms, assuming slit-shaped pores.

The morphology of the samples was studied by scanning electron microscopy (SEM) in an AURIGA FIB-FESEM microscope provided by Carl Zeiss SMT (Jena, Germany). The inorganic content in the catalysts was determined by thermogravimetric analysis (TGA) with a Mettler-Toledo TGA/DSC1 thermogravimetric analyzer (Greifensee, Switzerland). The TGA was carried out in air flow from 20 °C to 900 °C with heating rate of 10 °C min^−1^. The crystallinity of manganese oxide particles of all CK2-Mn-X-Y composites was investigated by X-ray diffraction Bruker D8 (Karlsruhe, Germany) advance with Cu Kα radiation.

The surface chemistry of the samples was obtained by X-ray photoelectron spectroscopy using ESCA 5701 from Physical Electronics (PHI, Chanhassen, MN, USA) system (equipped with MgKα anode, model PHI 04-548. X-ray source (hν = 1253.6 eV) and hemispherical electron energy analyzer. For the analysis of the XPS peaks, the C_1s_ peak position was set at 284.6 eV and used as reference to locate the other peaks. The XPS peaks were fitted using Gaussian–Lorentzian peak shapes and a Shirley background through the least squares method, utilizing XPS peaks 4.1.

### 2.3. Electrochemical Characterization 

The prepared CK2-Mn-X-Y composites were electrochemically characterized in a Biologic VMP Multichannel potentiostat (Grenoble, France) using a Rotating Ring-Disk Electrode (RRDE) (Metrohm AUTOLAB RDE-2, 3 mm Glassy Carbon tip, Utrech, Holland) where the sample was deposited as working electrode. The working electrode was prepared by depositing on the RRDE tip 20 µL of an ink, consisting of 5 mg of sample dispersed in 1 mL of a Nafion solution (1/9 *v*:*v* Nafion 5%/water), and dried under infrared radiation.

The activity for ORR was studied using the RRDE technique by using an Autolab electrochemical system, Metrohm (Utrech, Holland), associated with a compact potentiostat/galvanostat (PGSTAT101). Electrochemical measurements were carried out in a standard three-electrode electrochemical cell at room temperature. Using a Pt sheet as a counter electrode and an Ag/AgCl reference electrode. The electrolyte was a solution of KOH 0.1 M prepared in deionized water. Before each electrochemical measurement, the electrolyte was saturated with N_2_ or O_2_ by purging the necessary gas into the KOH 0.1 M solution at room temperature for 30 min. Cyclic voltammetry (CV) was collected in the KOH 0.1 M solution saturated with N_2_ or O_2_ in a range of 0.40 to −0.80 V at 50 mV s^−1^ with the electrode at a rotational speed of 1000 rpm. Linear scanning voltammetry (LSV) for the ORR was carried out with a potential range between 0.40 V and −0.80 at 50 mV s^−1^ with the electrode at rotational speeds of 500, 1000, 1500, 2000, 2500, 3000, and 3500 rpm.

The number of electrons transferred and H_2_O_2_ selectivity were calculated using Equations (5) and (6), which are calculated from the data obtained in the RRDE during the experiment [49,50]

(5) $$n=\frac{4\times I_{D}}{I_{D}+\frac{I_{R}}{N_{C}}}$$

(6) $$\%H_{2}O_{2}=\frac{200\times \frac{I_{R}}{N_{C}}}{I_{D}+\frac{I_{R}}{N_{C}}}$$

where $I_{D}$ and I_R_ are the disk and ring current, respectively, and N_C_ is the collection efficiency of the RRDE (0.249).

The current density (J_K_) was obtained by Koutecky–Levich equation (Equation (7)).

(7) $$\frac{1}{J}=\frac{1}{J_{K}}+\frac{1}{B\times w^{1/2}\;}$$

with $B=\left(0.62\right)n\times F\times A\times D^{2/3}\times v^{-1/6}\times C$, where F is Faraday’s constant (96,485,332 mC mol^−1^), n number of electrons transferred per oxygen molecule, A is the disc area of the RRDE (0.2475 cm^2^), D diffusion coefficient of oxygen (1.9 × 10^−5^ cm^2^ s^−1^), ν is kinematic viscosity (0.01 cm^2^ s^−1^), and, finally, C is the solubility of oxygen (1.2 × 10^−6^ mol cm^−3^) [51,52].

### 2.4. Electro-Fenton Processes

The electro-Fenton process was carried out using a standard three-electrode electrochemical cell with capacity for 150 mL of solution at room temperature. The TC concentration approached 40 mg L^−1^, using Na_2_SO_4_ [0.5 M] as a supporting electrolyte with continuous agitation. Potentiostat was maintained in potentiostatic mode at −0.6 V.

The working electrode was prepared by mixing 45 mg of CK2-Mn-X-Y with 8.5 mg of 60% PTFE, for subsequent drying at 100 °C for 12 h. Once dried, a paste was obtained that was deposited on a sheet of graphite (50 mg on each face). While the reference electrode was Ag/AgCl, and the counter electrode used was a platinum sheet. The working pH was 6.5, as is natural for the TC solution. TC concentrations in solution were determined by a UV–vis spectrophotometer at a wavelength of 356.5 nm.

## 3. Results and Discussions 

### 3.1. Morphological and Textural Characterization

#### 3.1.1. Morphology

**Figure 1:**
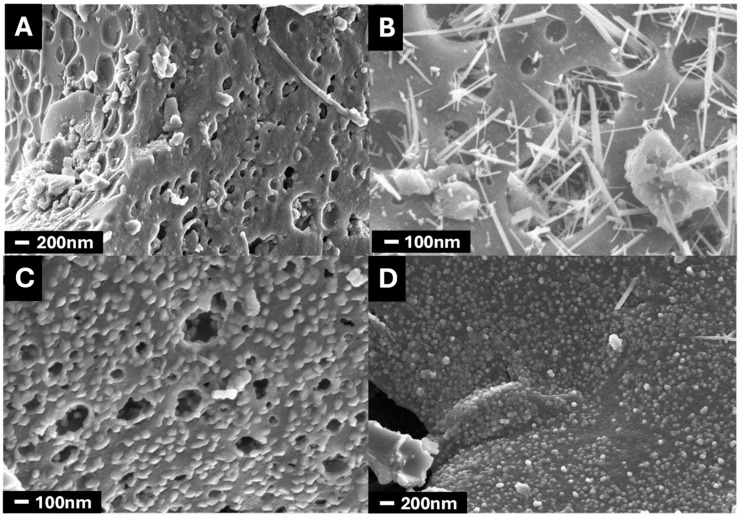
SEM microphotographs of CK2 (**A**), CK2-Mn-1-10 (**B**), CK2-Mn-2-10 (**C**), and CK2-Mn-3-10 (**D**).

The SEM micrographs (Figure 1) show the attack of the activator on the carbon surface. The microporous structure of the raw material is combined with the presence of mesopores formed by the reaction between KOH and carbon (A). After doping with manganese oxide, we can observe manganese oxide nanostructures that vary in morphology depending on the preparation method. Thus, we can see that in sample CK2-Mn-1-10, these nanostructures appear in the form of nanofilaments, whereas in samples CK2-Mn-2-10 and CK2-Mn-3-10, they appear in the form of spheres or hemispheres deposited on the bio-carbon support. This difference shows that the preparation method influences the final shape and size of the manganese oxide catalyst and consequently the electrocatalytic performance.

#### 3.1.2. The Porosity and Surface Area of the Composites

**Figure 2:**
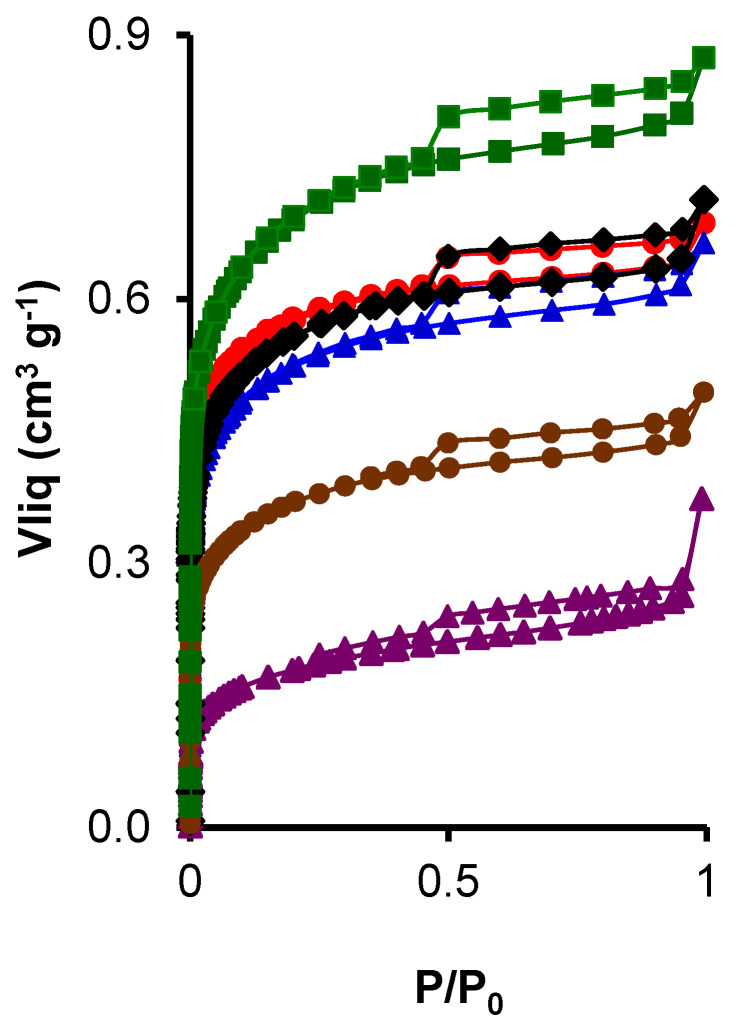
Isotherms N_2_ adsorption of samples CK2, 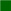; CK2-Mn-1-10, 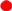; CK2-Mn-2-10, 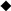; CK2-Mn-3-10, 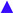; CK2-Mn-2-25, 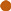; CK2-Mn-2-60, 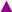.

Textural properties of the composites were examined by N_2_ adsorption–desorption isotherm measurements. Figure 2 shows the N_2_ adsorption–desorption isotherms of the CK2-Mn-X-Y samples. All samples show a hybrid-type I-IV isotherm, with a high N_2_ adsorption at low relative pressures and a hysteresis loop at intermediates ones, indicating the presence of micropores and mesopores [53]. Also, the samples CK2-Mn-3-10, CK2-Mn-1-10, and CK2-Mn-2-60 present a peak associated with capillary condensation. Note that the addition of Mn to the samples produces a blockage of the porosity in all cases; however, this porosity depletion depends on the final precursor decomposition treatment. From Figure 1, it is possible to observe that the H_2_O_2_ activation before carbonization (CK2-Mn-2-10) results in the better preservation of the porosity, due possibly to the chemical attack in the porous texture of the sample (CK2), which leads to the generation of new porosity. Finally, it is highlighted that the Mn amount directly affects the porosity; the higher the Mn content, the higher the porosity decrease, which is associated with the blockage of the pores of the carbon matrix by the manganese particles; this can be observed in the samples CK2-Mn-2 with different Mn charges (10, 25, and 60).

The B.E.T surface area (S_BET_), micropore surface area (S_micro_), mesopore surface area (S_DFT_), and pore volume (V_DFT_) of the CK2-Mn-X-Y samples are listed in Table 1.

The same results can be observed by analyzing Table 1. The original activated carbon presents a surface area as high as 1672 m^2^/g. This porosity decreases after the incorporation of Mn; however, this porosity decrease is less pronounced when the pristine carbon is treated with H_2_O_2_ before the manganese decomposition at 330 °C. The physical activation with CO_2_ results in a micropore volume lower than with the other methods (chemical activation) decreasing from 0.38 (CK2) to 0.29 cm^3^ g^−1^, indicating the blockage of the ultramicroporosity by the Mn nanoparticles or an opening of such porosity by the CO_2_ treatment, which is in agreement with the reported in the literature [54]. On the other hand, the decrease in narrow micropore volume and total micropore volume, of the samples CK2-Mn-2-25 and Ck-2-Mn-60, can be attributed to pore enlargement and blockage of these pores with Mn particles, which result in a decrease in B.E.T surface area, indicating a lower development of microporosity [55]. The sample CK2-Mn-3-10 has an L_0_(CO_2_) greater than 0.7 nm and a W_0_(N_2_) < W_0_(CO_2_), indicating a diffusional problem of N_2_, which translates into a bad activation degree, due to restrictions in the microporosity, while the other samples have W_0_(N_2_) > W_0_(CO_2_), which is typical of an adequate activation degree.

### 3.2. TGA 

**Figure 3:**
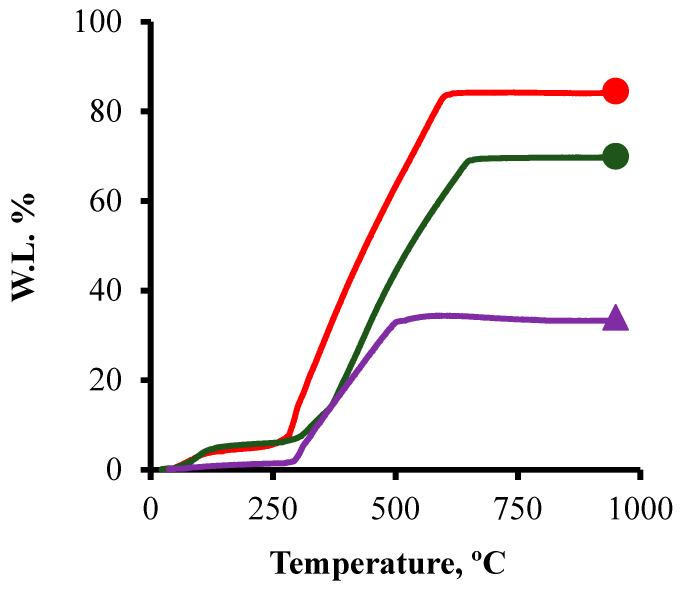
TGA analysis of samples CK2-Mn-2-10, 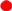; CK2-Mn-2-25, 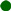; CK2-Mn-2-60, 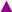.

Figure 3 shows the TGA analysis of the samples CK2-Mn-1-10, CK2-Mn-2-25, and CK2-Mn-2-60. From the final residual mass, the amount of manganese oxide was determined. The percentage of manganese oxide in the samples was 15.51, 30.02, and 66 wt.% for CK2-Mn-1-10, CK2-Mn-2-25, and CK2-Mn-2-60, respectively. The theoretical and TGA content of manganese in the samples are close, which indicates a good doping method.

### 3.3. X-ray Diffraction 

**Figure 4:**
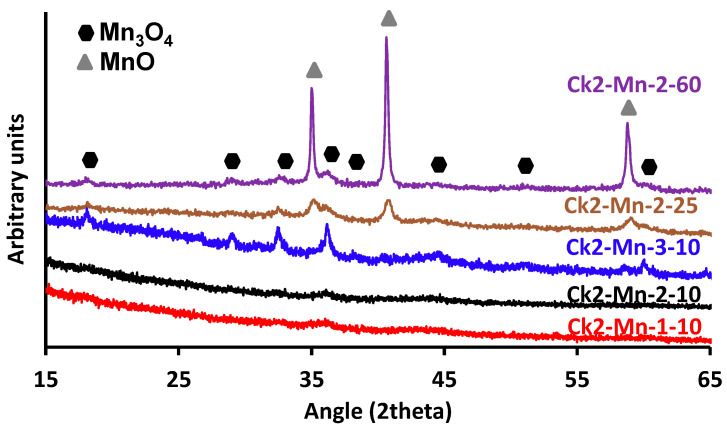
X-ray diffraction of the composites.

Figure 4 shows the XRD diffractograms of the CK2-Mn-X-Y samples, where we can observe different peaks associated with some species of Mn, indicating the presence of manganese in different oxidation states. It is possible to determine the peaks to approximately 18°, 29°, 32°, 36°, 38°, 44°, 58°, 60°, and 65°, associated with the planes (101), (112), (103), (211), (004), (220), (321), (224), and (400) characteristics of spinel structure Mn_3_O_4_ (CK2-Mn-3-10; ICSD card number 76088) [56,57,58]. While the peaks closer to 35°, 40°, and 59° correspond to the crystalline planes (111), (100), and (220) of MnO (CK2-Mn-3-60; ICSD card number 657304) [59,60,61]. The samples CK2-Mn-1-10 and CK2-Mn-2-10 present spectra corresponding to practically amorphous specimens, so raising the dispersion of manganese particles was better in these two materials. By increasing the manganese content, a new crystalline phase emerges, finding well-defined peaks of MnO crystalline phase form for the CK2-Mn-2-25 and CK2-Mn-2-60 samples.

From these results, it would be expected that better catalytic results will be obtained with the samples with 10% of Mn, due to the presence of Mn_3_O_4_, a species considered active for promoting ^·^OH generation by ORR [62].

## 4. Surface Chemistry of Composites

### 4.1. XPS Spectra

**Figure 5:**
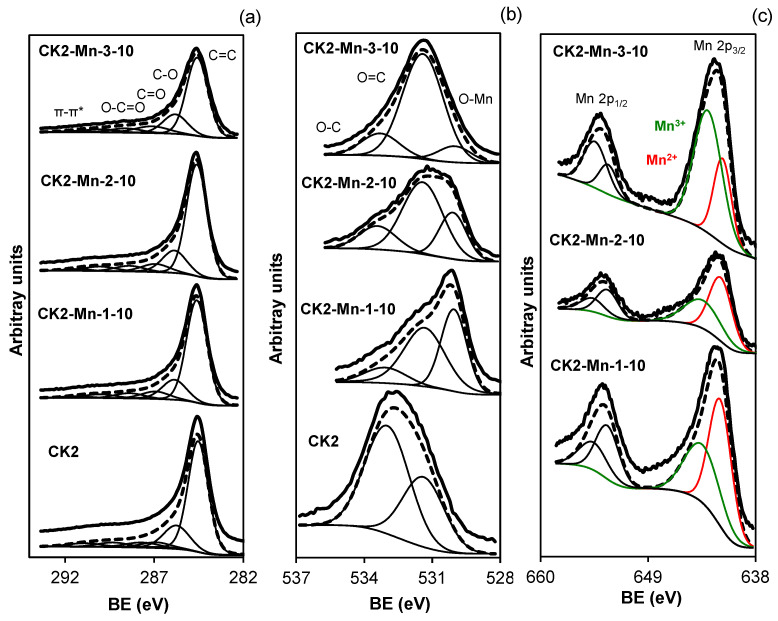
XPS spectra of (**a**) C_1s_, (**b**) O_1s_, and (**c**) Mn_2p_ for original activated carbon CK2 and samples prepared with different impregnation methods and 10% of manganese loading.

**Figure 6:**
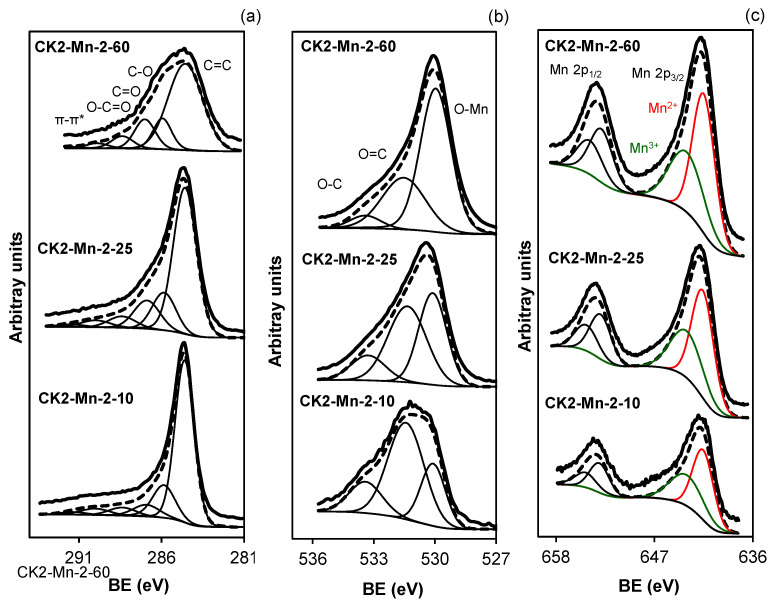
XPS spectra of (**a**) C_1s_, (**b**) O_1s_, and (**c**) Mn_2p_ for samples prepared with different amounts of manganese.

Figure 5 and Figure 6 show the XPS spectra for the signals C_1s_, O_1S_, and Mn_2p_. The surface composition of the samples was determined from the deconvolution of the XPS spectra, and the results are summarized in Table S1 (Supplementary Materials). The peaks obtained from the deconvolution of the C_1s_ spectrum were related to C=C (284.6 eV), C-O (285.7 eV), C=O (287 eV), O-C=O (288.4 eV), CO_2_ or π-π* bonds and plasmon (290 and 291.6 eV) [63,64,65,66,67]. The peaks from O_1s_, were deconvoluted in O-Mn (530.0 eV), O=C (531.4 eV), and O-C (533.4 eV) [68,69]. From Mn_2p_ the peaks detected were Mn^2+^ (641.6 eV) and Mn^3+^ (643.4 eV) [70,71,72,73,74].

From the results obtained, it can be concluded that all samples present similar composition profiles, with the same chemical species being found in all cases (see Table S1 Supplementary Materials). However, several significant facts are worth noting. The first one is the increase in the C peak width at 284.6 eV, which is indicative of an increase in the defects present in the graphitic crystals or a decrease in the size of the microcrystals, both facts will influence the electrical conductivity of the catalysts. Secondly, there is a noticeable increase in the O content when Mn is introduced into the catalysts, this is due to the oxidation produced by the reduction of Manganese acetate during the heat treatment. With respect to the Mn spectrum, two clearly differentiated species Mn^2+^ and Mn^3+^ are detected in all samples. However, a clear difference appears for sample CK2-Mn-3-10 for which the Mn^3+^/Mn^2+^ ratio is higher than for the rest of the samples, which is indicative of the formation of Mn_3_O_4_ due to the oxidation treatment carried out with H_2_O_2_, which agrees with the data obtained by XRD. For the rest of the samples, this ratio varies very little, between 0.64 and 0.71, indicating that part of the Mn^2+^ formed by the reduction treatment can be oxidized superficially to Mn^3+^ by exposure to air.

### 4.2. Electrochemical Characterization

**Figure 7:**
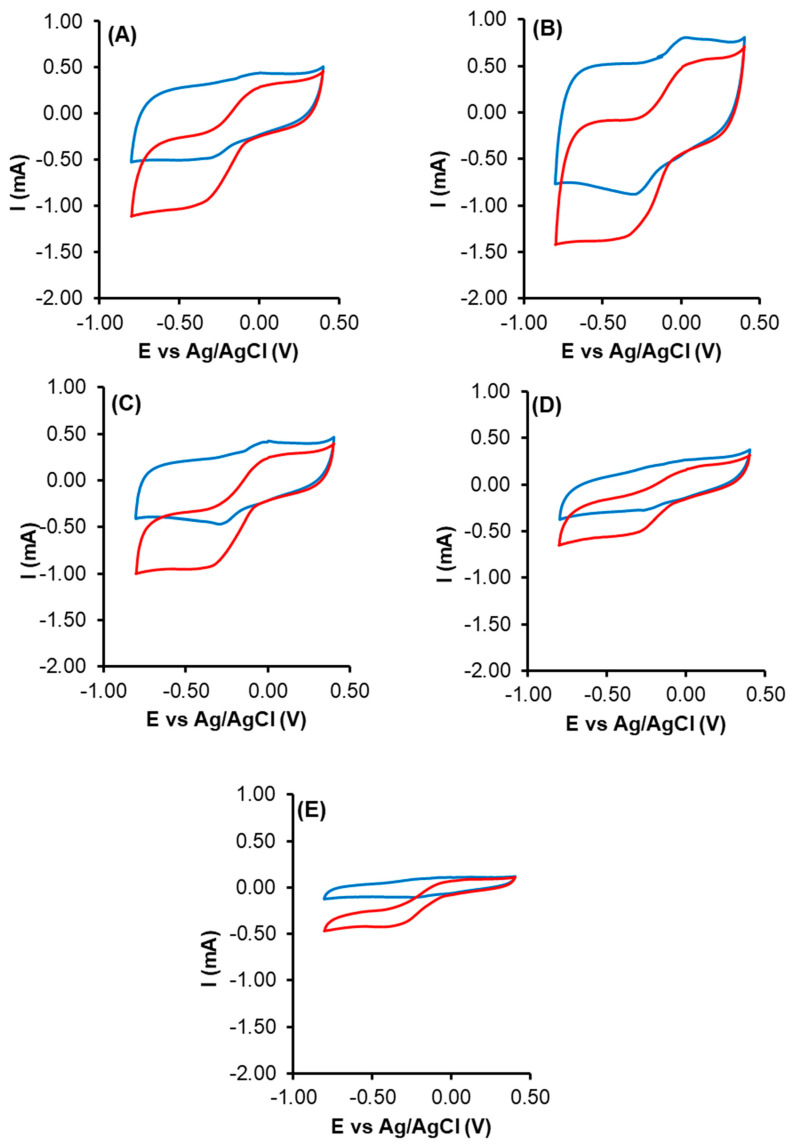
CV of (**A**) CK2-Mn-1-10, (**B**) CK2-Mn-2-10, (**C**) CK2-Mn-3-10, (**D**) CK2-Mn-2-25, (**E**) CK2-Mn-2-60 in N_2_ (blue) and O_2_ (red).

The CV results are shown in Figure 7, where is possible to observe that all samples have ORR activity. The sample CK2-Mn-2-10 showed the higher capacitance attributed principally to the type of porosity and large specific surface [75]. These better behaviors could be related to the improved hydrophilicity of the surface due to the increase in the oxygen surface groups and the presence of MnOx, which improves the electrolyte–surface contact favoring the diffusion of the ions, and the optimization of the pore structure by the presence of MnOx. This leads to a decrease in the resistance of the electrodes, enhancing their performance for energy storage.

Based on its chemical composition and textural characteristics, sample CK2-Mn-2-10 is expected to have better ORR activity and higher capacitance compared to the other samples. There is no visible OPR peak when the electrolyte is saturated with N_2_. In contrast, a well-defined ORR peak is observed in the O_2_-saturated 0.1 M KOH solution, demonstrating excellent electrocatalytic activity for ORR. More interestingly, all CV curves (both N_2_ and O_2_ saturated) show strong redox peaks, the peak at ca. 0 V vs. Ag/AgCl was attributed to Mn^2+^/Mn^3+^ redox because the area of the peak at ca. 0 V vs. Ag/AgCl increases with greater Mn concentration (Mn content changes from 10 to 60) [76], and especially with the amount of Mn^2+^ and Mn^3+^ present in the sample.

In this line, Table 2 shows that the highest J_K_ value was obtained with the pre-activated sample with H_2_O_2_ (CK2-Mn-2-10), which possibly is due in one part to the well-dispersed manganese phase and also to the highest surface area, which allows more current to pass into the matrix, for the same could explain the J_K_ value lower of CK2-Mn-2-25 and CK2-Mn-2-60. 

**Figure 8:**
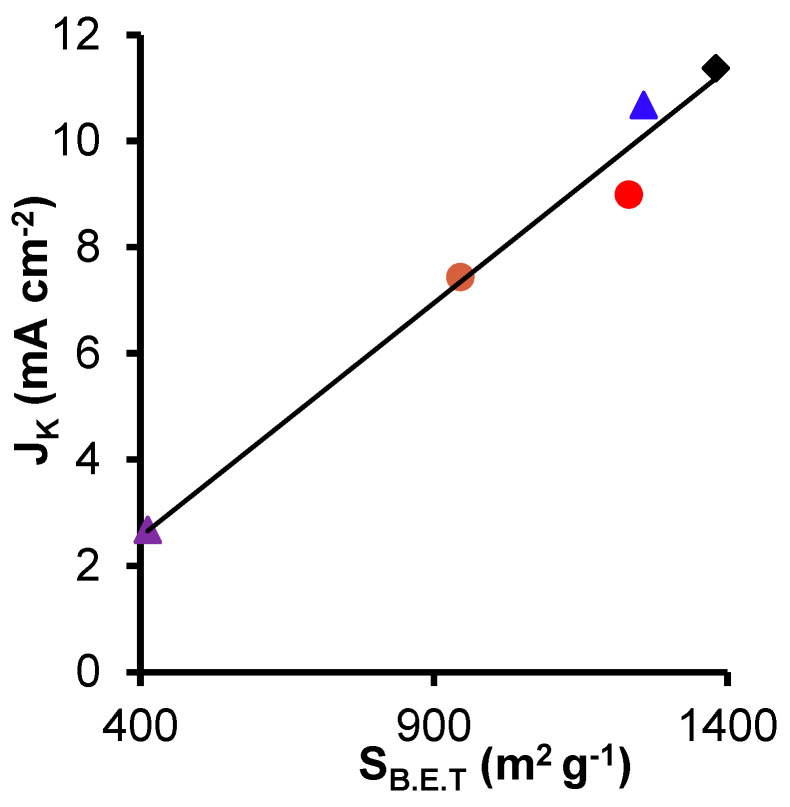
Correlation between the current density and the surface area B.E.T. of CK2-Mn-1-10, 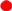; CK2-Mn-2-10, 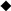; CK2-Mn-3-10, 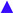; CK2-Mn-2-25, 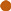; CK2-Mn-2-60, 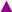.

**Figure 9:**
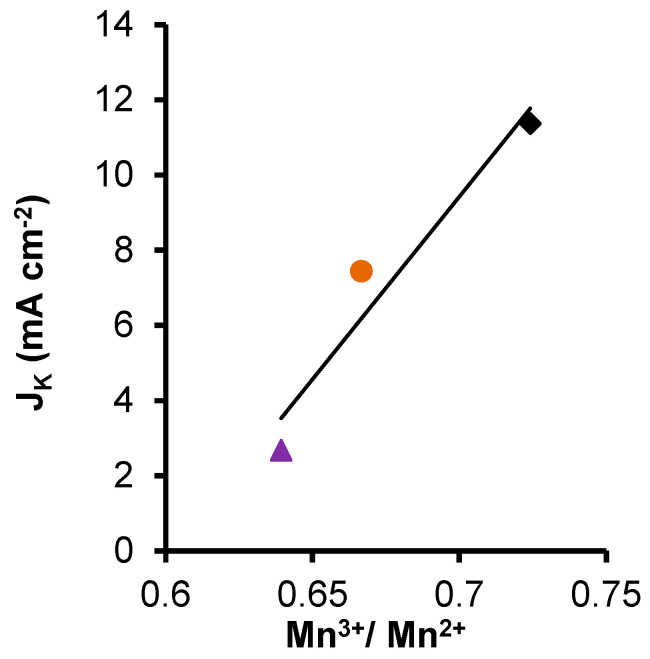
Correlation between the current density and the Mn^3+^/Mn^2+^ ratio of CK2-Mn-2-10, 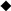; CK2-Mn-2-25, 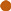; CK2-Mn-2-60, 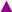.

Figure 8 and Figure 9 show the correlation between J_k_ and the Mn^3+^/Mn^2+^ amount and S_B.E.T._, respectively. The correlation coefficient of R^2^ was closer to 1 in S_B.E.T._ (0.98) than with Mn^3+^/Mn^2+^ amount (0.94), indicating that although the manganese can be an active site for the ORR, the current density is affected principally for the S_B.E.T_. [77]. In this case, we can say that physical activation gives as a result a higher surface area that is traduced in a better J_K_, which is an important parameter for the different electrochemical applications.

**Figure 10:**
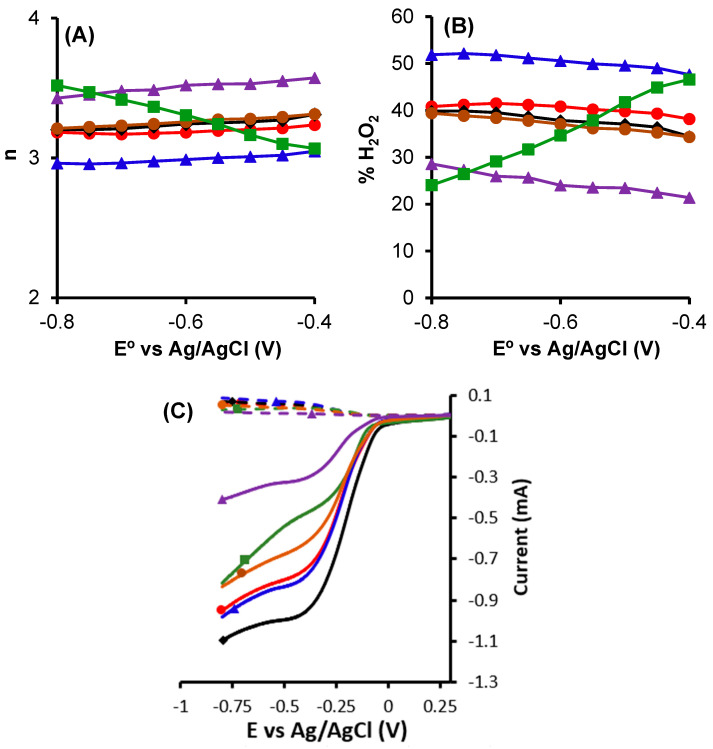
(**A**) Number of electrons transferred, (**B**) Selectivity to H_2_O_2_, (**C**) Current Ring and Disc of CK2, 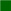; CK2-Mn-1-10, 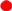; CK2-Mn-2-10, 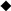; CK2-Mn-3-10, 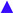; CK2-Mn-2-25, 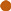; CK2-Mn-2-60, 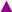.

Figure 10 shows the number of electrons transferred and selectivity to H_2_O_2_. Where it is possible to observe that the presence of Mn increases the number of electrons transferred, which is favorable for the ORR 4e^−^; however, for the case of H_2_O_2_ generation, it is necessary that the values are closer to two electrons, with a high selectivity to H_2_O_2_; for this, it is possible to suppose that the best catalyst for electro-Fenton is obtained by using the samples CK2-Mn-1-10 and CK2-Mn-3-10, which have higher amounts of manganese on the external surface area compared to the sample CK2-Mn-2-10:14.9 and 16.6, respectively, and also, a higher surfaces area compared to other samples with higher amounts of manganese CK2-Mn-2-25 and CK2-Mn-2-60. In this case, we can observe that both the amount of Mn and the surface porosity could be important parameters for the number of electrons transferred. Nevertheless, according to DRX results, with the increase from 10 to 60 wt.% of Mn on the same series, significant chemical changes occurred: sample CK2-Mn-2-10 only could show some Mn_3_O_4_ phases, while the presence of MnO appears in sample CK2-Mn-2-25 and in sample CK2-Mn-2-60 as the main phase. On the other hand, sample CK2-Mn-3-10, which shows higher and better-defined peaks of Mn_3_O_4_, is the one that produces a larger amount of H_2_O_2_ and shows an n-value closer to three.

### 4.3. Electro Fenton

Since the presence of an MnO phase does not favor the desired ORR performance, only samples with a 10 wt.% of Mn were checked in the electro-Fenton tests. In this way, Figure 9 shows the tetracycline degradation with the above-mentioned samples as well as with graphite only (an electrode-support material on which the prepared samples are pasted for the electro-Fenton tests). 

**Figure 11:**
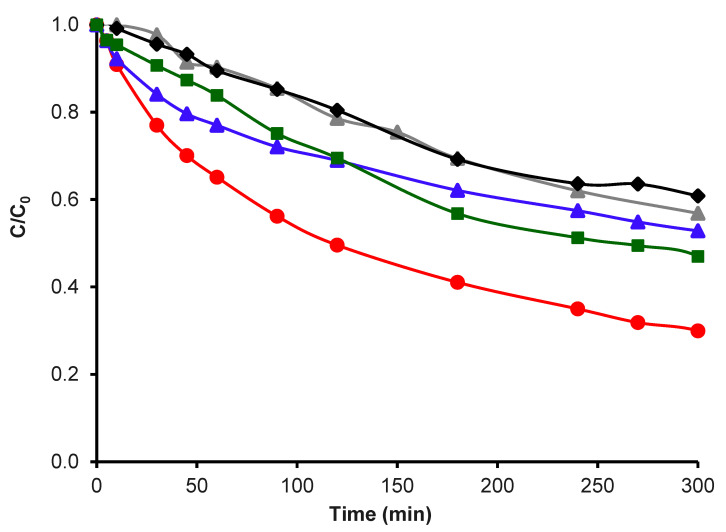
TC degradation by electro-Fenton with Graphite, 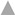; CK2, 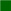; CK2-Mn-1-10, 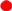; CK2-Mn-2-10, 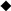; CK2-Mn-3-10, 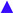.

Additionally, were made electro-Fenton tests in N_2_ with H_2_O_2_ and N_2_ without H_2_O_2_ (Figure 11), with the objective of evaluating the effect of hydrogen peroxide and of the support.

**Figure 12:**
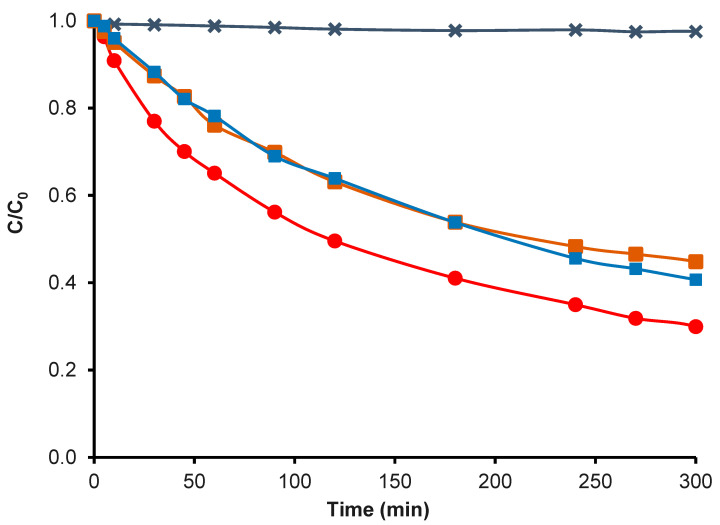
TC degradation by electro Fenton with CK2-Mn-1-10 in O_2_, 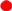; CK2-Mn-1-10 in Fenton traditional, 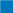; CK2-Mn-1-10 in N_2_ and H_2_O_2_, 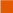; H_2_O_2_ without catalyst, 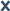.

The TC degradation in the presence only of O_2_ followed an order of CK2-Mn-1-10 > CK2 > CK2-Mn-3-10 > Graphite > CK2-Mn-2-10, whereby the sample CK2-Mn1-10 was used to evaluate its activity in presence of N_2_ with H_2_O_2_. The TC degradation in the presence of N_2_ showed a lower value compared with O_2_ (Figure 12), with this experiment having established that O_2_ presence has an important effect on the system, much more than the direct addition of H_2_O_2_ and obviously more than with only the presence of N_2_ in the total absence of O_2_ and H_2_O_2_.

These results could hide a probable three electrons ORR (Equation (8)) pathway with the direct formation of hydroxyl radicals (^·^OH), which enhances tetracycline degradation, as is observed in Figure 12 by comparing the effect of H_2_O_2_ addition vs. O_2_ bubbling [78].

(8) $$O_{2}+3e^{-}+2H^{+}\to {\;}^{\cdot }\mathrm{OH}+{HO}^{-}$$

However, it is not possible to dismiss the route by electro-Fenton traditional with the ORR pathway with two electrons with an initial reduction of O_2_ to H_2_O_2_ (Equation (9) [79].

(9) $$O_{2}+2e^{-}+{2H}^{+}\to H_{2}O_{2}$$

(10) $${H_{2}O}_{2}+e^{-}+H^{+}\to H_{2}O+{\;}^{\cdot }\mathrm{OH}$$

In this process, the Mn^3+^/Mn^2+^ redox cycle in the spinel structure is crucial since electrons can be provided. Moreover, the following way would be working in parallel:

(11) $${Mn^{2+}+H_{2}O}_{2}\to Mn^{3+}+{\;}^{\cdot }\mathrm{OH}+{HO}^{-}$$

(12) $$Mn^{3+}+e^{-}\to Mn^{2+}$$

However, with our results, it is proposed that the surface area B.E.T has a more significant effect in TC degradation (R^2^ > 0.6) by affecting the value of J_k_, which could be attributed to greater degree of access to the active sites.

**Figure 13:**
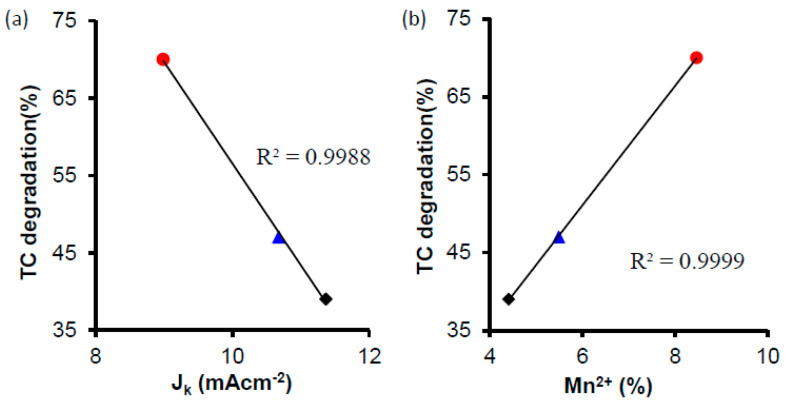
Correlation between the TTC degradation and the J_K_ value (**a**) and % of Mn^2+^ (**b**), of CK2-Mn-1-10, 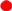; CK2-Mn-2-10, 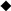; CK2-Mn-3-10, 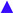.

In fact, it seems that a relationship between the Mn^2+^ values and tetracycline degradation occurs: CK2-Mn-1-10 (8.42 Mn^2+^; 70% TC removal), CK2-Mn-3-10 (5.48 Mn^2+^; 47% TC removal), and CK2-Mn-2-10 (4.40Mn^2+^; 39% TC removal) (Figure 13). The relevance of Mn^2+^ can be explained by the interaction between Mn^2+^ and H_2_O_2_, which can generate ^·^OH radicals (Fenton traditional), and by the possible direct generation of ^·^OH radicals (ORR 3e^−^ proposed) with Mn^3+^, which is proposed as active site for this route. We propose that a good relationship between the Mn^2+^ percentage and the surface area is key in the catalytic activity for degrading the tetracycline. So, we can conclude that the tetracycline degradation depends on J_k_, which depends on the surface area and the percentage of manganese, which interacts with H_2_O_2_, generating ^·^OH radicals. 

## 5. Conclusions

Two series of bio-carbon–manganese composites were prepared with bifunctional behavior in the electro-Fenton process: H_2_O_2_ generation from ORR, and hydroxyl radical formation. Moreover, the hydroxyl radicals can be formed in two different ways, (i) by H_2_O_2_ decomposition on Mn phases as heterogeneous Fenton, or (ii) by H_2_O_2_ reduction in one-pot way directly from the ORR following a pathway or three electrons. Moreover, the current density during the ORR is directly proportional to the surface area of these materials. The development of the Mn_3_O_4_ phase by a proper preparation method in these composites seems to be the clue that both mentioned processes can take place, as a consequence of the Mn^3+^/Mn^2+^ redox cycles that can occur in the spinel structure. This bifunctional behavior makes possible the antibiotic degradation in water solutions by a process in which H_2_O_2_ does not need to be added because it is in situ produced in such any way.

## Figures and Tables

**Table 1:** Textural characteristics of all the samples obtained by N_2_ adsorption isotherms at −196 °C and CO_2_ at 0 °C.

Samples		N_2_		CO_2_	
	S_BET_	W_0_	L_0_	W_0_	L_0_
	m^2^ g^−1^	cm^3^ g^−1^	nm	cm^3^ g^−1^	nm
CK2	1672	0.66	1.33	0.38	0.68
CK2-Mn-1-10	1232	0.50	1.32	0.29	0.67
CK2-Mn-2-10	1380	0.56	1.33	0.31	0.67
CK2-Mn-3-10	1257	0.50	1.31	0.87	1.28
CK2-Mn-2-25	945	0.37	1.33	0.22	0.82
CK2-Mn-2-60	412	0.16	1.40	0.10	0.73

**Table 2:** Current density J_k_ (mA cm^−2^).

Sample	J_K_ (mA cm^−2^)
CK2	7.39
CK2-Mn-1-10	8.99
CK2-Mn-2-10	11.37
CK2-Mn-3-10	10.68
CK2-Mn-2-25	7.44
CK2-Mn-2-60	2.68

## Data Availability

Data are contained within the article and supplementary materials.

## Supplementary Materials

The following supporting information can be downloaded at: https://www.mdpi.com/article/10.3390/nano14131112/s1, Table S1. Compositional characterization by XPS analysis.
