# Supplementary material for: Antibiotic Degradation via Fenton Process Assisted by a 3-Electron Oxygen Reduction Reaction Pathway Catalyzed by Bio-Carbon–Manganese Composites
Source: Nanomaterials (Basel). 2024 Jun 28;14(13):1112. doi: 10.3390/nano14131112 (PMC11243440; doi:10.3390/nano14131112)
Supplement: Supplementary file 1 [file nanomaterials-14-01112-s001.zip › nanomaterials-3030702-supplementary.pdf]

Supporting information

Antibiotic degradation via Fenton process assisted by a 3-electron oxygen reduction reaction pathway catalyzed by bio-carbon-manganese composites

Edgar Fajardo Puerto [a], Abdelhakim Elmouwahidi *[a], Esther Bailón-García [a], María Pérez-Cadenas [a,b], Agustín F. Pérez-Cadenas [a] and Francisco Carrasco-Marín [a]

[a.] UGR-Carbon - Materiales Polifuncionales Basados en Carbono, Dpto. Química Inorgánica - Unidad de Excelencia Química Aplicada a Biomedicina y Medioambiente - Universidad de Granada (UEQ-UGR), ES18071-Granada, España.

[b] Dpto. Química Inorgánica y Técnica, Facultad de Ciencias, UNED, Av. de Esparta s/n, Las Rozas de Madrid (Madrid), 28232, España.

* Correspondence: author: Abdelhakim Elmouwahidi, Email: aelmouwahidi@ugr.es

Table S1. Compositional characterization by XPS analysis

Sample	C_{1s} eV	FWHM eV	peak %	O_{1s} eV	peak %	O %	$Mn_{2p3/2}$ eV	peak %	Mn %	Mn^{3+}/Mn^{2+}
CK2	284.5	1.34	64	531.4	38	6.0				
	285.8		18	533.0	62					
	286.9		5							
	287.7		5							
	289.3		4							
	290.9		3							
CK2-Mn-1-10	284.6	1.37	67	530.0	39	11.9	641.6	58	14.6	0.70
	285.9		16	531.3	49		643.5	42		
	286.9		7	533.1	12					
	288.4		5							
	290.0		4							

	291.3		1							
CK2-Mn-2-10	284.6	1.37	66	530.1	25	8.8	641.6	58	7.6	0.72
	285.9		17	531.4	57		643.4	42		
	286.9		7	533.4	18					
	288.4		5							
	290.0		4							
	291.3		2							
CK2-Mn-3-10	284.6	1.41	63	530.0	9	18.3	641.7	31	17.7	2.25
	285.8		18	531.4	76		643.2	69		
	287.1		9	533.3	15					
	288.6		5							
	290.0		4							
	291.4		2							
CK2-Mn-2-25	284.6	1.66	61	530.1	40	25.6	641.6	61	32.9	0.66
	285.9		16	531.4	46		643.4	39		
	286.9		13	533.3	14					
	288.4		5							
	290.0		4							
	291.5		1							
CK2-Mn-2-60	284.6	2.45	64	530.0	63	35.5	641.5	60	52.8	0.64
	286.0		13	531.5	32		643.4	40		
	287.0		13	533.4	5					
	288.4		6							
	289.9		3							
	291.6		1							
